# Combining Ketamine, Brain Stimulation (rTMS) and Mindfulness Therapy (TIMBER) for Opioid Addiction

**DOI:** 10.7759/cureus.11798

**Published:** 2020-11-30

**Authors:** Basant Pradhan, Garrett Rossi

**Affiliations:** 1 Psychiatry, Cooper University Hospital, Camden, USA

**Keywords:** ketamine, neuromodulation, addiction psychiatry, opioid use disorders, tms, transcranial magnetic stimulation, mindfulness, mind-body medicine

## Abstract

Opioid addiction in the United States currently presents a national crisis despite availability of different treatments. Prior findings suggest that both repetitive transcranial magnetic stimulation (rTMS) and subanesthetic dose of ketamine are efficacious in patients with opioid use disorders (OUD) when administered in isolation. However, their therapeutic value may be undermined by varying clinical responses that tend to dissipate with treatment cessation. There has been no study to date that has used a combination of both for OUD, and there are still many unanswered questions with respect to both. TIMBER (Trauma Interventions using Mindfulness Based Extinction and Reconsolidation of memories) therapy attempts to alter the expression of emotionally charged memories such as traumatic memories, and has been successfully tried in chronic post-traumatic stress disorder (PTSD) and in combination with memory-altering pharmacotherapy like ketamine infusion. By a combination of extinction and reconsolidation of memory approaches, TIMBER works to not over-flood and/or retraumatize as is seen in other treatment approaches. TIMBER involves a balanced combination of both the memory extinction and memory reconsolidation approaches (rather than extinction-only approaches) which explains its superior efficacy in PTSD and benefit in substance use disorders.

## Introduction

Opioid addiction in the United States (US) currently presents a national crisis despite availability of different treatments. Over 2.1 million people were diagnosed with opioid use disorder (OUD) in 2016 [1]. Prior findings suggest that both repetitive transcranial magnetic stimulation (rTMS) and subanesthetic dose of ketamine are efficacious in patients with OUD when administered in isolation [1-4]. However, their therapeutic value may be undermined by varying clinical responses that tend to dissipate with treatment cessation. There has been no study to date that has used a combination of both for OUD, and there are still many unanswered questions with respect to both. Trauma Interventions using Mindfulness Based Extinction and Reconsolidation of memories (TIMBER) attempts to alter the expression of emotionally charged memories such as traumatic memories, and has been successfully tried in chronic post-traumatic stress disorder (PTSD) alone, or in combination with memory-altering pharmacotherapy like ketamine infusion [5-7]. By a combination of extinction and re-consolidation of memory approaches, TIMBER works to not over-flood and/or re-traumatize as is seen in other treatment approaches. At the present time, TIMBER has been shown to be efficacious in targeting trauma memories and their expressions in PTSD patients [6]. 

TIMBER uses all eight limbs of Yoga including the focused attention meditation and mindfulness meditation, the five-factor model of trauma or addiction experience, and integrates the principles of cognitive behavioral therapy (CBT) (mindfulness-based graded exposure therapy: MB-GET) with the neurobiology of emotionally charged traumatic or addiction memories. With respect to applying the translational model of TIMBER for drug addiction, the key steps are:

1. Inducing a mindful state (calm, alert but non-reactive) by use of a standardized meditation protocol (StaMP).

2. Creating drug-cues evoked controlled arousal response using a scripted brief narrative of the addiction experience by using the Buddha’s Five Factor Model (thoughts, feelings, memories, sensations/perceptions, and urges/impulses related to the addiction experience).

3. Using the mindfulness-based graded exposure therapy (MB-GET) and the TIMBER methods to decrease the arousal responses.

4. Using the already created mindful state to cognitively reprocess and reappraise the drug-related emotionally charged memories so that the drug-related emotionally charged memories are extinguished and de-conditioned from the cues and re-consolidated/updated as new memories.

TIMBER involves a balanced combination of both the memory extinction and memory reconsolidation approaches (rather than extinction only approaches) which likely explains its superior efficacies in PTSD and possibly in our future studies on drug addiction as well. The case series presented here is the extended application of the proof of concept model of TIMBER in three patients with chronic OUD.

## Materials and methods

The study is an open-label proof of concept study designed to test the feasibility and efficacy of ketamine, rTMS, and TIMBER in patients with OUD. The study included three patients all with a diagnosis of OUD. The primary outcome measure was percent reduction in cravings using the opiate use craving scale from baseline to the fifth rTMS session.

The following interventions were used:

1. Ketamine was given as a single infusion of 0.75 mg/kg weight-based dose capped at 745 mg total, administered over a 45 minute period. A one week washout period followed prior to the start of rTMS.

2. rTMS was performed for five sessions of 10 Hz and 3000 stimulation pulses applied to the right dorsolateral pre-frontal cortex (DLPFC) over one to two weeks.

3. Five sessions of TIMBER mindfulness-based therapy was carried out during the same two weeks period that rTMS was performed.

4. Home practice was then carried out two times daily with additional sessions on an as-needed basis whenever cravings were present.

Cravings were measured using the Opiate Craving Scale. Arousal responses were measured by a multi-system biofeedback bundle that comprised of real-time electroencephalogram (EEG), heart rate variability, and breath response pattern guided focused attention and mindfulness meditation practice (three minutes before rTMS, 21 minutes during, and three minutes after administration of rTMS).

## Results

In our open-label pilot study sample that consisted of three subjects with opiate use disorder (two with heroin use disorder and one with oxycodone use disorder), the participants rated their craving on the Opiate Craving Scale (OCS: scores range 0-30), five minutes before and five minutes after the rTMS stimulation [8]. They rated their level of mindfulness pre-infusion baseline and after completing five sessions of TMS+mindfulness using the Assessment Scale for Mindfulness Interventions (ASMI) [6,7].

In these three subjects, at baseline, the mean OCS was 23.6 (SD 4.2), which indicates a high level. The OCS reduced to 8.2 (SD 2.7) after five sessions of TMS and mindfulness. Similarly, at baseline, their mean ASMI was 28.45 (SD 9.61). After five sessions of TMS and mindfulness, the mean ASMI was 49. 67 (SD 7.72) indicating a significant increase in mindfulness (Table 1).

**Table 1:** Reduction In Opioid Craving and Increase In Mindfulness at Baseline and Session 5 OCS: Opiate Craving Scale, ASMI: Assessment Scale for Mindfulness Interventions

Patient Profile	Cravings at Baseline	Cravings at session Five	Craving Percent Decrease After 5 Sessions	Mindfulness at Baseline	Mindfulness at Session 5	Mindfulness percent Increase After 5- sessions	t-test P-Value (2-tailed), and 95% CI
Combined Patient Data	OCS: 23.66 +/- 3.21	OCS: 8.33 +/- 2.5	65.7%	ASMI: 29 +/- 3.6	ASMI: 49.33 +/- 7.37	41.21%	For OCS: P=0.002, t=6.5, 95% CI=8.8 to 21.85 For ASMI: P=0.01 T= 4.2, 95% CI= 33.48 to 7.18

## Discussion

Collectively all of these therapeutic interventions show promise as individual treatments for maintenance of abstinence in OUD, particularly improvement in cravings and increased motivation to quit. Significant long-term improvements in complete abstinence have been demonstrated with the use of ketamine following extended inpatient treatment [3,4], and ketamine reduced physiological response during opioid withdrawal [9]. However, these studies on the use of ketamine are limited by their use of low-dose ketamine as the comparison group rather than a true placebo. Repetitive transcranial magnetic stimulation (rTMS) is a noninvasive brain stimulation technique currently approved for major depressive disorder. rTMS is currently being pursued as a treatment for substance use disorder. Preliminary data looking at treatment with rTMS to the dorsolateral pre-frontal cortex (DLPFC) has shown reduction in cravings in alcohol, cocaine, and nicotine use disorders [2]. Single session rTMS studies have demonstrated that applying excitatory rTMS to the DLPFC can decrease cue-induced craving in nicotine, cocaine, and alcohol use disordered populations. The mechanism of rTMS success in treating addiction is thought to involve increased dopamine function in the shell region of the nucleus accumbuns [10].

Single session studies have only found small temporary reductions in craving, however more frequent sessions could lead to longer durations and greater reductions in cravings. The largest such clinical trial (n=130 smokers) demonstrated that 13 sessions of DLPFC rTMS resulted in six month tobacco abstinence rates of 33% [2]. Trauma memories lay at the core of the pathogenesis of PTSD. TIMBER therapy attempts to alter the expression of emotionally charged memories such as traumatic memories, and has been successfully tried in chronic PTSD alone or in combination with memory altering pharmacotherapy like ketamine infusion [5,7]. There are many similarities between the emotionally charged memories common in PTSD, and the emotionally charged memories associated with addiction. Exposure to environmental cues such as people, places, or drug paraphernalia lead to a state of arousal and strong emotional response that often results in relapse for patients attempting to maintain sobriety. The use of mindfulness-based graded exposure therapy (MB-GET) and the TIMBER methods to decrease the arousal responses will extinguish drug-related emotionally charged memories while replacing them with new more adaptive memories. TIMBER aims to restructure cognitions and emotions, which prevents reactivity of the underlying memories in trauma [5].

A number of important questions remain. It is unclear how each of these modalities will function in combination and in comparison to a true placebo group. It is unclear what role baseline levels of desire to quit, motivation for treatment, and prior periods of abstinence have on the achievement and maintenance of abstinence. The groups chosen for the ketamine studies were treatment-seeking and completed three months of residential treatment prior to treatment with ketamine infusion [3,4]. The abstinence rates at one- and two-year follow-up for ketamine are promising; unique genetic and socioeconomic factors must be considered [3,4]. To date there is limited work examining the effect of rTMS on craving in OUD. However, preliminary data indicates significant possibility of reduced craving with multiple sessions of rTMS. While TIMBER methods have been successfully used to treat PTSD, this method has yet to be applied to substance use disorders on a mass scale. TIMBER involves a balanced combination of both the memory extinction and memory reconsolidation approaches (rather than extinction only approaches) and has possible implications for substance use disorder. 

## Conclusions

Combination therapy with ketamine, rTMS and TIMBER is feasible in patients with OUD. Although an open-label proof of concept study, the data suggests that the combination therapy reduces craving, promotes abstinence, and reduces the amount used in patients with OUD. Combination therapy allows patients to be actively involved in their care by engaging in meditative therapy-based techniques that directly result in a calmer state of mind. This protocol allows concurrent implementation of three individually effective interventions in combination for a compounding synergistic effect. 
